# Aortic root dimensions as a correlate for aortic regurgitation’s severity

**DOI:** 10.1007/s10554-021-02337-6

**Published:** 2021-07-07

**Authors:** Jan-Per Wenzel, Elina Petersen, Julius Nikorowitsch, Jessica Müller, Tilo Kölbel, Hermann Reichenspurner, Stefan Blankenberg, Evaldas Girdauskas

**Affiliations:** 1 Department of General and Interventional Cardiology, University Heart and Vascular Center Hamburg, Hamburg, Germany; 2 German Center for Cardiovascular Research (DZHK), Partner Site Hamburg/Kiel/Luebeck, Hamburg, Germany; 3 Department of Cardiovascular Surgery, University Heart and Vascular Center Hamburg, Hamburg, Germany; 4 Epidemiological Study Center, Hamburg, Germany; 5 Department of Vascular Medicine, German Aortic Center Hamburg University Heart and Vascular Center, Hamburg, Germany

**Keywords:** Aortic root, Echocardiography, Sinotubular junction, Aortic annulus, Aortic regurgitation, Hamburg City Health Study

## Abstract

To evaluate the prevalence of aortic regurgitation (AR) and associations between the individual aortic root components and AR severity in the general population. The study included the first 10,000 participants of the population-based Hamburg City Health Study (HCHS) of whom 8259 subjects, aged 62.23 ± 8.46 years (51.3% females), enrolled 2016–2018, provided echocardiographic data. 69 subjects with bicuspid valves and 23 subjects with moderate/severe aortic stenosis were excluded. Aortic root dimensions were measured using state-of-the-art cardiac ultrasound, including the aortic annulus, sinus of Valsalva, sinotubular junction (STJ), and ascending aorta, in diastole and systole. The distribution of AR was: 932 (11.4%) mild, 208 (2.5%) moderate, and 20 (0.24%) severe. Patients with moderate or severe AR were predominantly male at advanced age who had hypertension, coronary artery disease, atrial fibrillation, and renal dysfunction. Increasing AR severity correlated with higher absolute and indexed aortic root diameters (e.g., end-diastolic sinus of Valsalva for no-mild-moderate-severe AR in mm ± standard deviation: 34.06 ± 3.81; 35.65 ± 4.13; 36.13 ± 4.74; 39.67 ± 4.61; p < 0.001). In binary logistic regression analysis, all aortic root components showed significant associations with moderate/severe AR. Mid-systolic STJ showed the strongest association with moderate/severe AR (OR 1.33, 95% confidence interval 1.25–1.43, p < 0.001). AR was prevalent in 14.2%, of whom 2.8% showed moderate/severe AR. All assessed aortic root diameters correlated with the prevalence and severity of AR. STJ diameter had the strongest association with moderate/severe AR possibly reflecting the pathophysiological impact of an increasingly dilated STJ in the context of an ageing aorta.

**Supplementary Information:**

The online version contains supplementary material available at 10.1007/s10554-021-02337-6.

## Introduction

The aortic root is a complex and dynamic entity, varying in size proportionally to height, weight, and age. The interplay of its four different parts, the aortic annulus (AoAn), sinus of Valsalva (SoV), sinotubular junction (STJ), and the proximal ascending aorta (AscAo), is crucial for aortic valve (AV) competence. Along with a dramatic decrease of rheumatic heart disease in the Western world, aortic root dilatation evolved into the predominant cause of aortic regurgitation (AR) [1–4]. However, AR is a multifactorial valvular disease and the correlation between the extent of aortic root dilatation and the severity of AR remains controversial [2, 3]. Notably, most previously published studies correlating AR with aortic root enlargement focused on the aortic root as one entity. Accordingly, little is known about the role of the individual components of the aortic root and their timepoint of measurement during the heart cycle in relation to AR, which is crucial to understand in the field of AV repair surgery. Furthermore, given major improvements in spatial and temporal resolution of 2-dimensional and color Doppler transthoracic echocardiography (TTE), most previous studies investigating AR prevalence and its correlation with aortic root diameter are rather outdated [2, 5–7].

Hence, first we aimed at providing up-to-date data on the prevalence of AR as assessed by state-of-the-art TTE in the general population. Second, we investigated the correlation between AR severity and each individual part of the aortic root measured systematically in end-diastole and mid-systole.

## Methods

### Study setting

The study population derived from a sample of 10,000 consecutive, at random selected participants from the Hamburg City Health Study (HCHS, www.hchs.hamburg) who underwent TTE. As previously described, the HCHS is a single-center, prospective, long-term, population-based cohort study [8]. HCHS aims to evaluate the interaction of socioeconomic risk factors, modern imaging techniques, physiological measurements, and clinical variables. All measurements were conducted between 2016 and 2018 during a one-day baseline visit at the HCHS Epidemiological Study Center Hamburg-Eppendorf, Germany according to the published study protocol [8]. Demographics and clinical parameters were assessed by standardized interviews conducted by specifically trained medical professionals following standard operating procedures as well as self-reported questionnaires [8]. Blood samples were withdrawn under fasting conditions and all subjects underwent biomarker quantification including NT-proBNP. After application of the exclusion criteria (1) incompletely recorded images or insufficient image quality of TTE for standardized measurements (1741) (2) moderate/severe aortic stenosis (23) or (3) bicuspid valve disease (69), 8167 subjects were included in the study. The study protocol was approved by the local ethics committee (PV5131, Medical Association Hamburg) and the HCHS steering board. All participants gave written informed consent. The investigation conforms with the principles outlined in the Declaration of Helsinki.

### TTE image acquisition and analysis

TTE examinations were performed and analyzed by professional cardiologists and sonographers (technicians) at the baseline visit on dedicated ultrasound machines (Siemens Acuson SC2000 Prime, Siemens Healthineers, Erlangen, Germany) following standard operating procedures at the HCHS Epidemiological Study Center Hamburg-Eppendorf, Hamburg, Germany [8]. All TTE standard views were assessed in 2-dimensional echocardiography, including a 3-dimensional four-chamber view for chamber quantification. For continuous quality assessment, every 100th TTE exam was analyzed twice by an ESC TTE certified cardiologist. Qualitative and quantitative image analyses were performed using an off-line workstation with the commercially available and established Siemens syngo SC2000 software (Siemens syngo SC 2000 Version 4.0, Siemens Healthineers, Erlangen, Germany) in agreement with the current recommendations of the American Society of Echocardiography (ASE) and the European Association of Cardiovascular Imaging (EACVI) [9, 10]. Left sided volumes and ejection fraction (LVEF) were calculated using from two-dimensional and three-dimensional loops using the method of disks summation. Left-sided diameters were measured in parasternal long-axis view. Mitral inflow pattern was assessed in apical four-chamber view by placing pulsed-wave (PW) Doppler sample volume between mitral leaflet tips. PW tissue Doppler imaging (TDI) e’ velocity was measured in apical four-chamber view by placing the sample volume at the lateral and septal basal regions. Tricuspid annular plane systolic excursion (TAPSE) was obtained by M-mode echocardiography in the apical four-chamber view. Right ventricular fractional area change was assessed in a right ventricular focused four-chamber view. Left atrial global peak systolic strain was measured in apical four-chamber view by velocity vector imaging averaging global peak strain of all segments of the left atrium.

### Aortic root and valve assessment

For aortic root assessment, a zoomed transthoracic parasternal long axis view was recorded at breath hold [10]. Systematic measurements of the aortic root were performed perpendicular to the proximal aorta axis in end-diastole (ED) as well as in mid-systole (MS) including the following: The (a) AoAn, (b) SoV, (c) STJ, and (d) AscAo (at 2 cm range from the STJ) were measured perpendicular to the proximal aorta axis. AoAn, STJ and AscAo were measured using the inner-edge to inner-edge convention while the SoV was measured using the leading-edge to leading-edge convention.

AV morphology (i.e., bicuspid vs. tricuspid) and potential degenerative changes were assessed in the parasternal long axis, AV short axis and apical views. Aortic stenosis and regurgitation were assessed according to current ESC guidelines [11]. An in-depth description of valve assessment is provided in the supplements.

### Statistical analysis

Continuous variables are given as mean ± standard deviation (SD). Categorical variables are given as absolute numbers and percentage of participants. Multiple group comparisons of different severities of AR were assessed by analysis of variance (ANOVA) for continuous variables and chi-squared test (*χ*^2^) for categorical variables. BSA was calculated using the DuBois formula. Aortic root measurements were divided by BSA to obtain indexed measurements. Correlation of these indexed measurements with AR severity as well as the ratio of sinotubular junction and aortic annulus were presented as boxplots using ANOVA to draw multiple group comparisons.

AR was dichotomized into “no AR” and “moderate/severe AR” for logistic regression analyses. We constructed a model including all aortic root measurements. However, substantial multicollinearity between measures of aortic root was detected using the variance inflation factor (VIF). Therefore, we constructed separate models for all eight aortic root measurements. Age, sex, BSA, hypertension, and diabetes were pre-defined as relevant confounders and controlled for in all aortic root models. Results of logistic regression were corrected post-hoc using the Holm method and are depicted by odds ratios (OR) and corresponding 95% confidence intervals (CI). Differences were considered statistically significant at a two-sided p-value level of 0.05 after post-hoc correction. All statistical analyses were performed using R (version 3.6.2). A list of the used packages and versions can be found in the appendix.

## Results

**Fig. 1:**
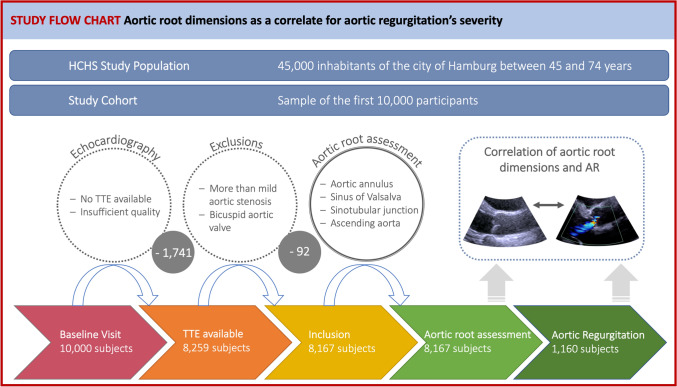
Study flow-chart. From a total of 8259 subjects providing echocardiographic data, 92 were excluded due more than mild aortic stenosis or bicuspid aortic valve. Consequently, 8167 subjects were included in the study analysis. Of those, 1160 subjects suffered from aortic regurgitation. The aortic root was systematically measured in end-diastole and mid-systole and the correlation between aortic root dimensions and the severity of aortic regurgitation was calculated. *AR* aortic regurgitation, *HCHS* Hamburg City Health Study, *TTE* transthoracic echocardiography

The analyzed study population included 8167 HCHS subjects of the first 10,000 HCHS participants, with 4191 female (51.3%) and a mean age of 62.23 ± 8.46 years (range 45–74 years) (Table 1; Fig. 1). 23 subjects were excluded due to moderate/severe AS and 69 due to bicuspid aortic valve morphology. While 7007 (85.8%) subjects were free of AR, 932 (11.4%) showed mild, 208 (2.5%) moderate and 20 (0.3%) severe AR. Patients with moderate or severe AR showed predominantly male sex, a higher age, and had more hypertension, CAD, atrial fibrillation, hypercholesterolemia, NT-proBNP elevation, and renal dysfunction compared with patients with no AR. Weight, height, heart rate, BSA, Glucose, triglycerides, hsCRP, hemoglobin, and diabetes did not show significant intergroup differences. The use of ACE inhibitors, angiotensin receptor blockers, beta-blockers, diuretics, and statins was more common in subjects with moderate/severe AR. Echocardiography revealed slightly lower left ventricular systolic function and larger left-sided cavities as well as a higher E/e’ ratio in subjects with moderate/severe AR compared to subjects without AR (Table 2). 7 subjects suffered from moderate/severe AR combined with mild AS. The prevalence of moderate/severe MR increased with AR severity.

**Table 1:** Baseline Characteristics of the study population stratified by AR severity

	No AR (n = 7007)	Mild AR (n = 932)	Moderate/Severe AR (n = 228)	p-value
Demographics and biological data				
Age, years	61.6 ± 8.4	66.0 + 7.8	67.3 ± 7.4	< 0.001
Male	3369 (48.1)	482 (51.7)	125 (54.8)	0.019
Weight, kg	78.6 ± 16.2	78.1 ± 15.3	77.5 ± 16.5	0.423
Height, cm	171.3 ± 9.5	171.2 ± 9.3	170.3 ± 9.9	0.359
BSA, m^2^	1.9 ± 0.2	1.9 ± 0.2	1.9 ± 0.2	0.34
Systolic blood pressure, mmHg	138.4 ± 19.2	140.9 ± 19.6	143.5 ± 18.4	< 0.001
Heart rate, bpm	69.8 ± 11.2	69.1 ± 10.5	69.6 ± 11.9	0.208
Current smoking	1438 (20.6)	114 (12.3)	32 (14.1)	< 0.001
NYHA, II/III	542 (8.6)	84 (10.0)	20 (10.5)	0.267
Comorbidities				
Hypertension	4292 (64.7)	638 (70.3)	171 (78.1)	< 0.001
Diabetes	546 ( 8.5)	68 ( 7.7)	17 (7.9)	0.728
Coronary artery disease	291 (5.9)	45 (6.9)	23 (14.9)	< 0.001
Atrial fibrillation	341 (5.4)	75 (8.9)	28 (13.4)	< 0.001
Peripheral artery disease	207 ( 3.2)	41 ( 4.9)	8 (4.1)	0.042
Medication				
Beta-blockers	1114 (16.7)	180 (20.0)	51 (23.3)	0.003
Diuretics	152 (2.3)	28 (3.1)	8 (3.7)	0.15
Statines	1132 (17.0)	182 (20.3)	53 (24.2)	0.002
ACE/AT-I-antagonists	1356 (20.4)	207 (23.1)	63 (28.8)	0.003
Laboratories				
LDL, mg/dl	121.0 [96.0, 146.0]	118.0 [93.0, 143.0]	119.0 [96.0, 150.0]	0.058
GFR, ml/min	86.3 [75.5, 94.7]	83.1 [71.9, 90.7]	81.7 [71.4, 88.2]	< 0.001
NT-proBNP, ng/l	77.0 [43.0, 141.0]	100.0 [54.3, 179.5]	136.0 [79.0, 228.0]	< 0.001
hsCRP,mg/l	0.12 [0.06, 0.26]	0.12 [0.06, 0.24]	0.12 [0.06, 0.26]	0.428
Glucose, mg/dl	92.0 [86.0, 100.0]	92.0 [96.0, 99.0]	93.0 [87.0, 100.0]	0.562

Data are given as n (%) or mean ± standard deviation, p-value for intergroup differences

*ACEi* angiotensin-converting enzyme inhibitor, *AR* aortic regurgitation, *ARB* angiotensin receptor blocker, *BMI* body mass index, *BSA* body surface area, *CAD* coronary artery disease; *hsCRP* high sensitivity C-reactive protein, *GFR* glomerular filtration rate, *NT-proBNP* N-terminal pro-B-type natriuretic peptide, *NYHA* New York Heart Association

**Table 2:** Echocardiographic findings of the study population stratified by AR severity

	No AR (n = 7007)	Mild AR (n = 932)	Moderate/Severe AR (n = 228)	p-value
Concomitant valvular disease				
Mild AS	86 (1.3)	23 (2.8)	7 (3.4)	< 0.001
Moderate/severe MR	189 (2.3)	74 (7.9)	19 (8.3)	< 0.001
Moderate/severe TR	623 (8.9)	193 (20.7)	46 (20.2)	< 0.001
Echocardiographic data				
LVEF 2D, %	58.5 ± 5.2	58.3 + 5.0	57.6 ± 5.6	0.045
LVEF 3D, %	59.6 ± 6.8	59.2 ± 6.6	58.3 ± 6.8	0.178
LVEDD, mm	47.7 ± 5.2	47.9 ± 5.3	48.9 ± 6.4	0.004
LVEDV, ml	114.9 ± 31.8	108.4 ± 30.6	118.9 ± 34.6	< 0.001
TAPSE, mm	24.4 ± 4.3	23.8 ± 4.4	23.4 ± 4.2	0.001
RV FAC, %	43.2 ± 8.0	43.7 ± 7.7	42.7 ± 8.4	0.217
IVSD, mm	9.9 ± 1.7	10.3 ± 1.8	10.7 ± 1.9	< 0.001
E/e ‘	7.6 ± 2.1	7.8 ± 2.4	8.5 ± 2.9	< 0.001
LAVI, ml/m^2^	27.5 ± 8.4	27.9 ± 8.9	30.2 ± 10.8	< 0.001
LA Global Peak strain, %	39.8 ± 14.4	38.5 ± 15.4	37.0 ± 14.5	0.014

Data are given as n (%) or mean ± standard deviation, p-value for intergroup differences. All subjects with moderate/severe aortic stenosis or bicuspid valves were excluded from the analysis

*AS* aortic stenosis, *IVST* interventricular septum thickness, *LA* left atrial, *LAVI* left atrial volume index, *LVEDD* left ventricular end-diastolic diameter, *LVEDV* left ventricular end-diastolic volume, *LVEF* left ventricular ejection fraction, *MR* mitral regurgitation, *MS* mitral stenosis, *RV FAC* right ventricular fractional area change, *TAPSE* tricuspid annular peak systolic excursion, *TR* tricuspid regurgitation

**Fig. 2:**
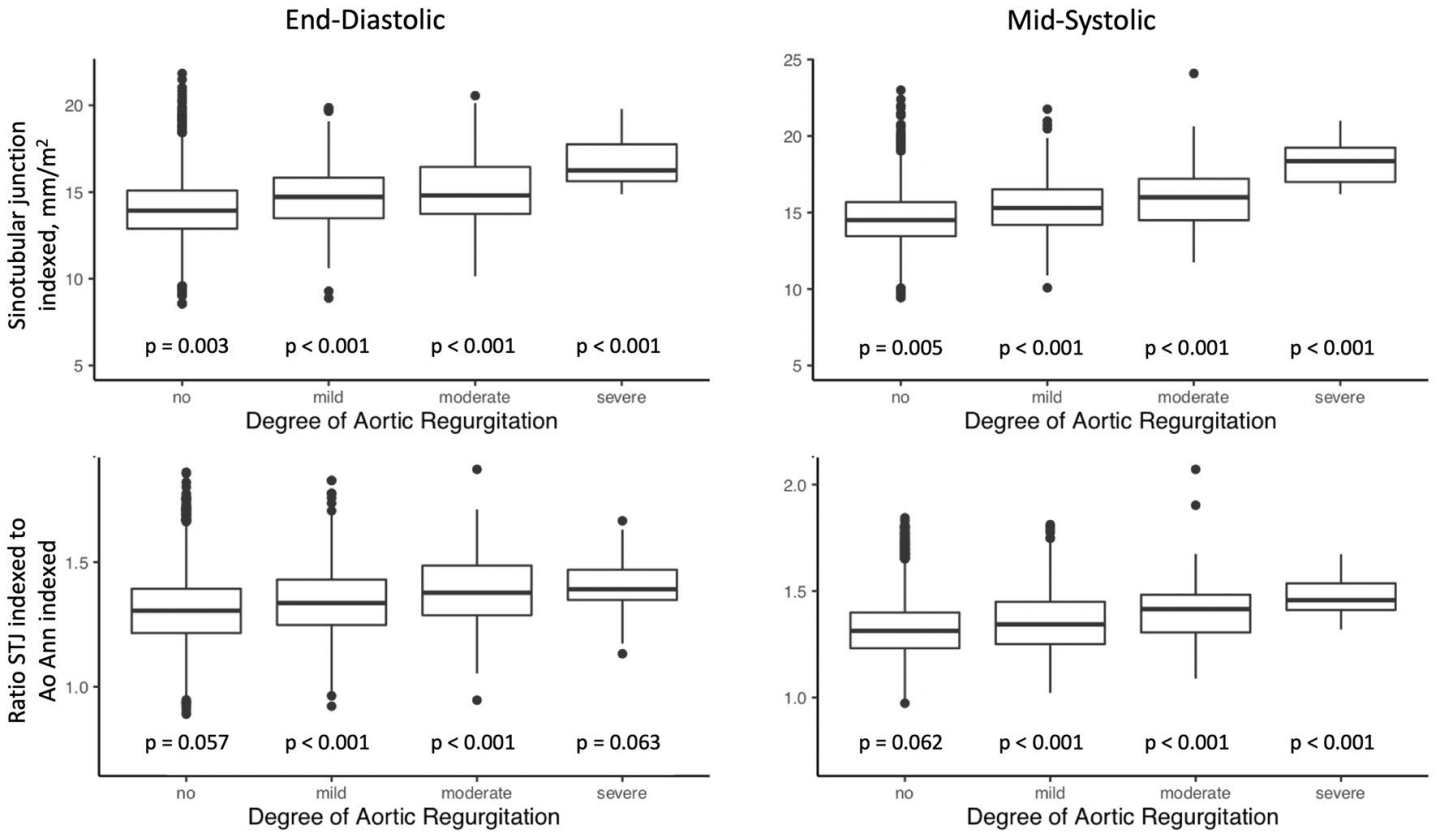
Correlation of AR severity with the sinotubular junction indexed to BSA as well as the ratio of sinotubular junction indexed and aortic annulus indexed to BSA. *p-*values for inter-group differences. *Ao Ann* Aortic annulus, *BSA* body surface area, *STJ* sinotubular junction

**Fig. 3:**
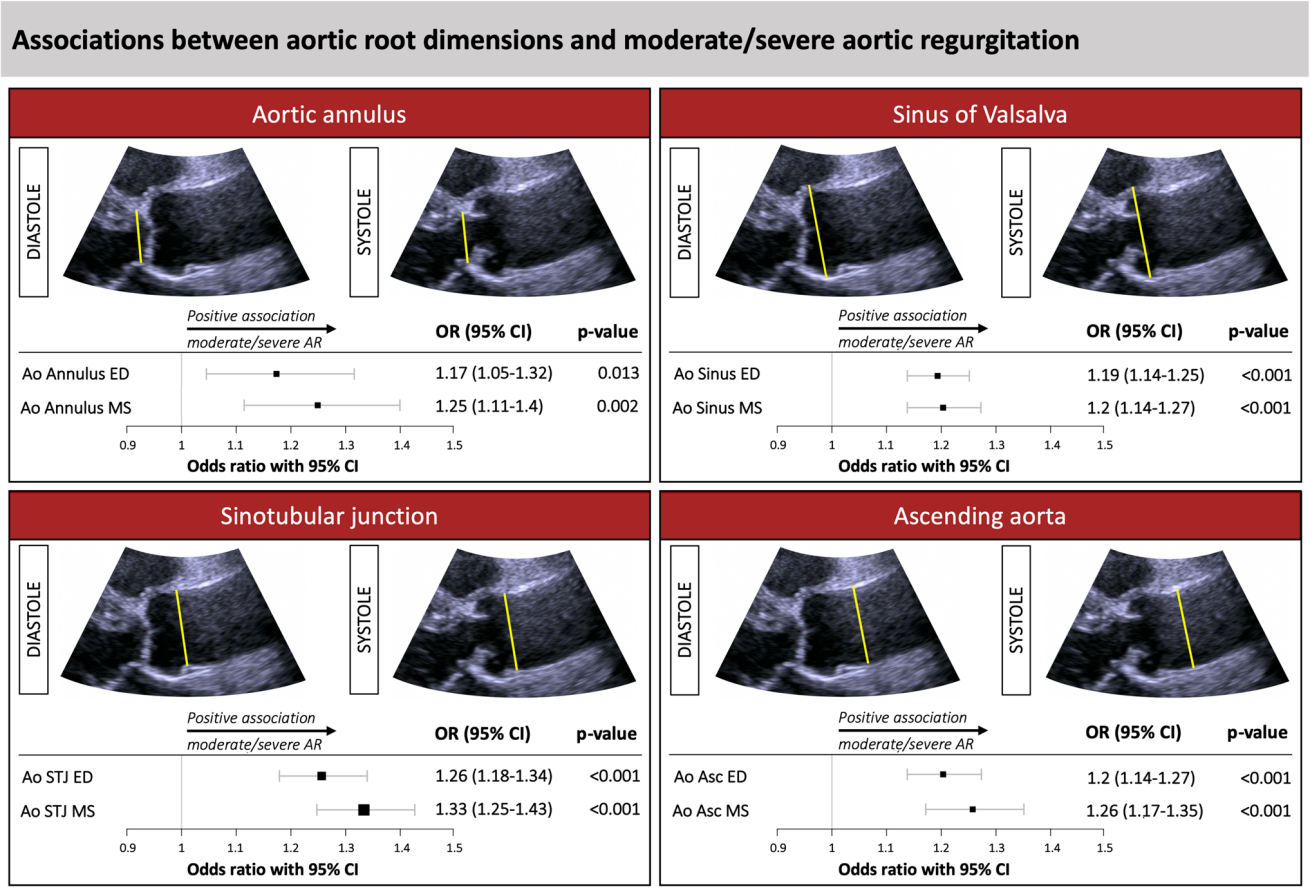
Associations between aortic root dimensions and moderate/severe aortic regurgitation derived from multivariate logistic regression analysis. Odds ratios derived from multivariate logistic regression analysis adjusted for age, sex, body surface area, hypertension, and diabetes with Holm corrected p-values. Squares and horizontal lines represent odds ratios and 95% confidence intervals. *Ao* aortic, *AR* aortic regurgitation, *Asc* ascending, *CI* confidence interval, *ED* end-diastolic, *MS* mid-systolic, *OR* odds ratio, *STJ* sinotubular junction

Inter- and intraobserver reproducibility of echocardiographic aortic root assessment, derived from the measurements of a random sample of 100 exams measured by three different observers, were remarkably high for all variables, as shown by intraclass correlation coefficients ranging from 0.92 to 0.99 (Supplemental Table 1). Systolic were greater than diastolic measurements of the aortic root. Both the absolute and indexed diameters of aortic root components showed the lowest values in the no AR group and the highest values in the severe AR group (Table 3; Fig. 2). In binary logistic regression, age was significantly associated with AR prevalence (OR 1.08, 95% CI 1.06–1.10, p < 0.001). Furthermore, both in univariate as well as in multivariate logistic regression analysis, adjusted for age, sex, BSA, hypertension, and diabetes, significant associations were detected for all end-diastolic and mid-systolic aortic root variables with mild as well as with moderate/severe AR, even after correcting for multiple testing (Table 4, Supplementary Table 2, Fig. 3, Supplementary Fig. 1 and 2). The exclusion of subjects with mild AS did not change the reported associations with aortic root diameters (Supplementary Table 3). The strongest association for the correlation with moderate/severe AR was found for MS STJ (OR 1.33, 95% confidence interval 1.25–1.43, p < 0.001).

**Table 3:** Aortic root diameters absolute (mm) and indexed to body surface area (mm/m^2^) in relation to AR severity

	None (n = 7007)	Mild AR (n = 932)	Moderate AR (n = 208)	Severe AR (n = 20)	p-value
Absolute					
ED Ao annulus	20.33 ± 1.71	20.65 ± 1.84	20.50 + 2.01	22.26 ± 1.91	< 0.001
MS Ao annulus	20.96 ± 1.76	21.35 ± 1.87	21.29 ± 1.98	22.19 ± 1.85	< 0.001
ED Ao sinus	34.06 ± 3.81	35.65 ± 4.13	36.13 ± 4.74	39.67 ± 4.61	< 0.001
MS Ao sinus	35.02 ± 3.82	36.15 ± 4.16	37.16 ± 4.69	40.88 ± 3.68	< 0.001
ED Ao STJ	26.53 ± 3.07	27.70 ± 3.25	28.33 ± 3.63	31.22 ± 3.27	< 0.001
MS Ao STJ	27.63 ± 3.24	29.01 ± 3.62	30.07 ± 3.80	33.65 ± 3.81	< 0.001
ED Asc Ao	29.94 ± 3.77	30.96 ± 4.11	32.56 ± 4.65	36.03 ± 3.43	< 0.001
MS Asc Ao	29.94 ± 3.51	30.99 ± 3.81	32.57 ± 4.12	35.67 ± 3.37	< 0.001
Indexed to BSA					
ED Ao annulus	10.79 ± 1.09	10.97 ± 1.08	11.03 ± 1.15	12.18 ± 1.25	< 0.001
MS Ao annulus	11.09 ± 1.08	11.32 ± 1.05	11.35 ± 1.20	12.00 ± 1.15	< 0.001
ED Ao sinus	18.01 ± 1.99	18.86 ± 2.06	19.18 ± 2.38	21.39 ± 1.75	< 0.001
MS Ao sinus	18.54 ± 2.04	19.23 ± 2.10	19.69 ± 2.38	22.30 ± 1.63	< 0.001
ED Ao STJ	14.03 ± 1.66	14.68 ± 1.77	14.99 ± 2.00	16.75 ± 1.55	< 0.001
MS Ao STJ	14.65 ± 1.73	15.42 ± 1.84	15.98 ± 2.10	18.28 ± 1.62	< 0.001
ED Asc Ao	15.79 ± 2.08	16.40 ± 2.17	17.10 ± 2.42	19.56 ± 2.40	< 0.001
MS Asc Ao	15.82 ± 1.96	16.48 ± 1.89	17.26 ± 2.04	19.41 ± 1.29	< 0.001

Data are given as mean ± standard deviation, p-value for intergroup differences

*AR* aortic regurgitation, *Asc* ascending, *Ao* Aortic/Aorta, *BSA* Body surface area, *ED* end-diastolic, *MS* mid-systolic, *STJ* sinotubular junction

**Table 4:** Odds ratios and 95%-CIs derived from univariate and multivariate regression analysis for the associations of aortic root measurements with moderate/severe AR

	Univariate		Multivariate	
	OR [95% CI]	p-value	OR [95% CI]	p-value
End-diastolic aortic root measurements				
Ao Ann ED	1.11 [1.02–1.21]	0.016	1.17 [1.05–1.32]	0.013
Age			1.09 [1.06–1.11]	< 0.001
Male sex			1.15 [0.72–1.85]	1.000
BSA			0.21 [0.07–0.67]	0.070
Hypertension			1.54 [1–2.42]	0.331
Diabetes			0.89 [0.56–1.34]	1.000
Ao Sinus ED	1.16 [1.12–1.2]	< 0.001	1.19 [1.14–1.25]	< 0.001
Age			1.07 [1.04–1.09]	< 0.001
Male sex			0.81 [0.52–1.27]	1.000
BSA			0.17 [0.06–0.46]	0.006
Hypertension			1.76 [1.19–2.68]	0.063
Diabetes			0.83 [0.53–1.22]	1.000
Ao STJ ED	1.24 [1.17–1.3]	< 0.001	1.26 [1.18–1.34]	< 0.001
Age			1.08 [1.05–1.11]	< 0.001
Male sex			0.93 [0.58–1.49]	1.000
BSA			0.2 [0.06–0.62]	0.049
Hypertension			1.5 [0.98–2.35]	0.360
Diabetes			0.91 [0.57–1.38]	1.000
Ao Asc ED	1.2 [1.15–1.26]	< 0.001	1.2 [1.14–1.27]	< 0.001
Age			1.07 [1.04–1.1]	< 0.001
Male sex			0.86 [0.5–1.47]	1.000
BSA			0.27 [0.07–1]	0.206
Hypertension			1.68 [0.97–3.09]	0.360
Diabetes			0.9 [0.52–1.45]	1.000
Mid-systolic aortic root measurements				
Ao Ann MS	1.16 [1.07–1.26]	< 0.001	1.25 [1.11–1.4]	0.002
Age			1.08 [1.05–1.1]	< 0.001
Male sex			1.01 [0.65–1.55]	1.000
BSA			0.26 [0.09–0.74]	0.070
Hypertension			1.66 [1.12–2.54]	0.132
Diabetes			0.85 [0.55–1.24]	1.000
Ao Sinus MS	1.17 [1.12–1.22]	< 0.001	1.2 [1.14–1.27]	< 0.001
Age			1.08 [1.05–1.11]	< 0.001
Male sex			0.74 [0.44–1.27]	1.000
BSA			0.2 [0.06–0.68]	0.070
Hypertension			1.71 [1.07–2.83]	0.209
Diabetes			0.9 [0.53–1.41]	1.000
Ao STJ MS	1.26 [1.19–1.32]	< 0.001	1.33 [1.25–1.43]	< 0.001
Age			1.08 [1.04–1.11]	< 0.001
Male sex			0.57 [0.32–1.04]	0.671
BSA			0.15 [0.04–0.61]	0.070
Hypertension			2.01 [1.16–3.68]	0.137
Diabetes			0.98 [0.54–1.62]	1.000
Ao Asc MS	1.23 [1.16–1.3]	< 0.001	1.26 [1.17–1.35]	< 0.001
Age			1.08 [1.04–1.12]	< 0.001
Male sex			0.73 [0.38–1.42]	1.000
BSA			0.2 [0.04–1]	0.206
Hypertension			1.4 [0.76–2.74]	0.905
Diabetes			1.05 [0.52–1.89]	1.000

P-values were corrected for multiple testing using the post-hoc Holm method

*Ao* aortic, *AR* aortic regurgitation, *Asc* ascending, *BSA* body surface area; *CI* confidence interval, *ED* end-diastolic, *MS* mid-systolic, *OR* odds ratio, *STJ* sinotubular junction

## Discussion

The present study analyzes standardized, state-of-the-art echocardiographic aortic root measurements in association with the severity of aortic regurgitation in a large sample of the population-based HCHS. Major findings include that AR is common in the general population and that the size of all parts of the aortic root are associated with moderate/severe AR. Of the different aortic root parts, MS STJ size showed the strongest correlation with moderate/severe AR.

### Prevalence of aortic regurgitation (AR)

The overall prevalence of AR in the population-based HCHS cohort, aged 45 to 74 years, was 14.2%, while the rate of moderate/severe AR was 2.8%. Subjects with moderate/severe AS or bicuspid valves were excluded from our analysis. Previously published studies on AR prevalence at the populational level reported very heterogeneous results [12–17]. AR prevalence ranged between 1 and 19%, depending on the baseline characteristics of the analyzed study cohort as well as on the methodology of echocardiographic evaluation. Most previously published studies demonstrated a significant association between age and AR prevalence [3, 13, 15]. While the overall prevalence of AR in younger populations is considerably low, it rises with ageing [3, 12, 13]. In line with this finding, we showed a significant association between age and AR prevalence. Furthermore, in several studies, including the Framingham Cohort Study, AR prevalence was associated with male sex [15, 18]. Nevertheless, other population-based studies did not find sex-associated differences in AR occurrence [3, 16, 17]. Our study showed in univariate analysis a significantly higher number of male subjects in the AR group, especially in those presenting with a moderate and severe AR. However, when controlling for age, BSA, and hypertension, this association did not remain statistically significant. Thus, the predominance of AR in male subjects might be primarily due to the higher prevalence of classical risk factors in males. Besides age and sex distribution, the improvement of echocardiographic technology might be causal for the relatively high AR prevalence in our study cohort since several of the mentioned studies are rather outdated. In particular, the high amount of mild AR cases might reflect a greater ability of current-era echocardiography equipment to detect small regurgitant jets. Furthermore, other comparable community based studies, as the OxVALVE Population Cohort Study (PCS), excluded subjects with known pre-existing valvular heart disease, resulting in a slightly lower rate of AR [19]. In summary, AR is a frequent finding at the populational-level, while its prevalence varies according to the analyzed study population and the utilized echocardiographic equipment.

### Aortic root size and AR

In the present study, all mid-systolic and end-diastolic aortic root diameters were larger with increasing severity and prevalence of AR. A significant correlation between aortic root size and AR has been previously demonstrated by several research groups [1–3, 7, 16, 20]. The AV is mounted in the complex structure of the aortic annulus that is tightly integrated into the functional unit of the aortic root. Therefore, dilatation of the entire aortic root or of its separate components may result in AV annulus dysfunction and subsequent development of AR. However, previous studies focused predominantly on the aortic root as one entity, without analyzing the separate components or the timepoint of measurement within the cardiac cycle. Therefore, we aimed to address this issue by examining all integral aortic root parts in systole and diastole. In adjusted binary logistic regression analysis, all individual parts of the aortic root were associated with moderate/severe AR. Mid-systolic measurements showed a tendency towards a stronger correlation with AR when compared to end-diastolic measurements. According to current recommendations by the ASE and EACVI, end-diastole is the proposed timepoint for all aortic measurements, except the AoAn [9]. Still, limiting the assessment solely to end-diastolic measurements does not comply with the complex interplay of the aortic root with pressure changes during the cardiac cycle which ensures AV integrity. In this regard, our data suggest a correlation of the same magnitude between AR and mid-systolic as compared to end-diastolic aortic root diameters. This finding can be explained by the fact that HCHS participants were at moderate to advanced age and had a “normal” tricuspid AV. These selection criteria resulted in an exclusion of most subjects with a dilated AoAn, who frequently present at a young age (i.e., congenital connective tissue disorders) and those with congenital AV lesions (i.e., bicuspid AV). Previous data demonstrated that the age-dependent increase in diameter is more prevalent in STJ as compared to AoAn [21]. Therefore, the likely explanation for the strongest correlation between AR severity and STJ diameter is given by the fact that our middle-aged /older study cohort with tricuspid AV undergoes a progressive STJ dilatation due to ageing-dependent remodeling, while those subjects with primarily dilated AoAn are predominantly excluded. The functional impact of STJ dilatation on the prevalence and the severity of AR results from the radial displacement of AV commissures creating a central coaptation defect with consecutive AR. The normalization of STJ diameter by surgical STJ annuloplasty or replacement of the ascending aortic aneurysm corrects the commissural displacement and is followed by improved AV competence.

Several previous studies focused on the definition of cut-off values of aortic root size which would be associated with AR occurrence [22]. Roman et al. and Seder et al. defined a threshold of 4.5 cm and 4.3 cm for the SoV diameter which were associated with a 100% prevalence of AR [1, 2]. Both studies used M-Mode echocardiography limiting the translation of these findings into the HCHS study design. Nonetheless, we were able to confirm and augment previous findings by demonstrating a linear correlation between aortic root dimensions and AR prevalence as well as an association between aortic root size and AR severity. However, due to the functional impact of several AV components on AV function and the development of AR (i.e., aortic cusps, AoAn, STJ) a simplified use of aortic root size to predict AR severity is rather counterproductive. The focus should rather be on the separate components of AV complex that determine AV function and their interaction.

### Limitations

The results of this study are influenced by the subjects included. Our study cohort represents the first 10.000 subjects of the HCHS. Most of the subjects were middle-aged and free of symptoms of cardiovascular disease. Therefore, the translation into other populations and utilization in the clinical setting is limited. Only a small proportion of the overall cohort showed severe AR. Although our study is derived from a large sample, most subjects showed mild AR, which led to an underrepresentation of the moderate/severe group. The degree of association of the different aortic root components with AR severity could vary considerably according to patients’ baseline characteristics. The presented regression analysis was adjusted for all detectable confounding variables. Nevertheless, we cannot exclude that other relevant interacting variables were not considered. The pathogenetic mechanism by which STJ diameter is associated with AR was not addressed. Whether AR itself induced the aorta to dilate or aortic root dilatation led to regurgitation was not answered. Most importantly, our study was not designed to investigate the causality of AR, which is not possible due to cross-sectional study design, but to evaluate the correlation between aortic root diameters and AR severity.

## Conclusion

In a large population-based sample, AR prevalence was 14.2%, with 2.8% being moderate/severe AR. Higher aortic root diameters at all levels were significantly associated with the prevalence and severity of AR. Of those, mid-systolic STJ had the strongest association with moderate/severe AR, reflecting its pathophysiological role in the context of an ageing aorta. Clinicians should closely monitor aortic root dilatation in the context of AR severity. Special focus should be paid to STJ dilatation as it showed the strongest association with a moderate/severe AR. Further validation in a broader, prospective population-based sample to evaluate the prognostic role of dilated aortic root diameters in the context of AR is needed.

## Supplementary Information

Below is the link to the electronic supplementary material.

Supplementary file1 (DOCX 141 kb)

## Data Availability

The data underlying this article cannot be shared publicly due to *the privacy of individuals that participated in the study.* The data will be shared on reasonable request to the corresponding author.

## Abbreviations

ACEi: Angiotensin-converting enzyme inhibitor
Asc: Ascending
Ao: Aorta/aortic
An: Annulus
AR: Aortic regurgitation
ARB: Angiotensin receptor blocker
ASE: American Society of Echocardiography
AV: Aortic valve
BMI: Bodyss mass index
BSA: Body surface area
CRP: C-reactive protein
EACVI: European Association of Cardiovascular Imaging
ED: End-diastolic/diastole
ESC: European Society of Cardiology
GFR: Glomerular filtration rate
HCHS: Hamburg City Health Study
IMT: Intima media thickness
IVST: Interventricular septum thickness
LAVI: Left atrial systolic volume indexed to body surface area
LVEDD: Left ventricular end-diastolic diameter
LVEDV: Left ventricular end-diastolic volume
LVEF: Left ventricular ejection fraction
MS: Mid-systolic/systole
NT-proBNP: N-terminal pro-B-type natriuretic peptide
NYHA: New York Heart Association
SoV: Sinus of Valsalva
STJ: Sinotubular junction
TAPSE: Tricuspid annular peak systolic excursion

## Code availability

Will be shared on reasonable request. List of used R packages: forestplot (1.10); ggpubr (0.2.4); gridExtra (2.3); reshape2 (1.4.3); survival (3.1-8); tableone (0.10.0); tidyverse (1.2.1)

## Declarations

### Illustrations and figures

The authors do hereby declare that all illustrations and figures in the manuscript are entirely original and do not require reprint permission.
