# Supplementary material for: Aortic root dimensions as a correlate for aortic regurgitation’s severity
Source: Int J Cardiovasc Imaging. 2021 Jul 7;37(12):3439–49. doi: 10.1007/s10554-021-02337-6 (PMC8604845; doi:10.1007/s10554-021-02337-6)
Supplement: Supplementary file 1 — Supplementary file1 (DOCX 141 kb) [file 10554_2021_2337_MOESM1_ESM.docx]

**13. Supplements**

**13.1. Aortic valve assessment**

Aortic stenosis was morphologically defined by a reduced aortic cusp motion. Functional measurements (i.e., peak jet velocity and mean transvalvular pressure gradient measurements) were obtained in a 5-chamber-view using continuous-wave (CWD) and pulsed-wave Doppler (PWD) examinations. Aortic valve area (AVA) was calculated by continuity equation. Moderate or severe AV stenosis was defined as peak jet velocity ≥3,0 m/s, mean transvalvular pressure gradient ≥20 mmHg, and AVA ≤0,85 cm^2^/m^2^. Aortic regurgitation was assessed by color Doppler-based measurements of the vena contracta (VC), proximal jet width to LVOT diameter ratio and CWD-based calculation of the pressure half time (PHT). VC was quantified in parasternal long axis view immediately before the regurgitant orifice, perpendicular to the direction of the jet, after reducing color sector size to maximize frame rate and optimization of color gain and scale. CWD measurements were performed in 5-chamber-view after optimization of Doppler beam alignment perpendicular to the jet direction. PHT was obtained by measuring the slope of the CWD AR signal. PHT was only considered if the jet was centric and the CWD signal was complete without any disruptions. Furthermore, the proximal jet width to LVOT diameter ratio was assessed by measuring the maximum color jet diameter in diastole at the junction of LVOT and AoAn precisely below the AV divided by LVOT diameter. The suprasternal view was not performed. The PISA method was not performed.

The classification was based on an integration of the following parameters: (1) mild AR when the VC width was <3 mm, PHT >500 ms, jet width/LVOT diameter <25%, (2) moderate AR when the VC width was ≥3 and <6 mm, PHT 200-500 ms, and jet width/LVOT diameter 25-65%, (3) severe AR when the VC width was ≥6 mm, PHT <200 ms, jet width/LVOT diameter >65%, LV enlargement.

A total of three cardiologists performed the assessment of AR severity. All moderate and severe AR ratings as well as a random sample of 10% of all mild AR ratings were verified by an ESC TTE certified cardiologist.

**13.2. Reproducibility of aortic root measurements**

**Supplementary Table 1. Reproducibility of the aortic root measurements.**

|  |  | Interobserver  ICC (95%-CI) | Intraobserver  ICC (95%-CI) |
| --- | --- | --- | --- |
| End-diastolic  measurements | Aortic annulus  Sinus of Valsalva  Sinotubular junction  Ascending aorta | 0.96 (0.95-0.96)  0.99 (0.99-0.99)  0.98 (0.98-0.98)  0.98 (0.98-0.98) | 0.92 (0.87-0.95)  0.99 (0.99-1.00)  0.99 (0.98-0.99)  0.99 (0.98-0.99) |
| Mid-systolic  measurements | Aortic annulus  Sinus of Valsalva  Sinotubular junction  Ascending aorta | 0.96 (0.96-0.96)  0.99 (0.99-0.99)  0.99 (0.99-0.99)  0.99 (0.99-0.99) | 0.96 (0.93-0.98)  0.99 (0.99-1.00)  0.99 (0.98-0.99)  0.99 (0.98-0.99) |

Abbreviations: CI = confidence interval, *ICC = intraclass correlation coefficient.*

Supplementary Table 2: Odds ratios and 95%-CIs for univariate and multivariate association of aortic root measurements with no vs. mild AR

|  | Univariate | | Multivariate | |
| --- | --- | --- | --- | --- |
|  | OR [95%-CI] | p-value | OR [95%-CI] | p-value |
| End-diastolic aortic root measurements | | | | |
| Ao Ann ED | 1.11 [1.06-1.16] | <0.001 | 1.13 [1.06-1.2] | 0.001 |
| Age |  |  | 1.07 [1.06-1.09] | <0.001 |
| Male sex |  |  | 0.89 [0.7-1.13] | 1.000 |
| BSA |  |  | 0.96 [0.54-1.68] | 1.000 |
| Hypertension |  |  | 0.82 [0.67-0.99] | 0.372 |
| Diabetes |  |  | 0.88 [0.64-1.2] | 1.000 |
| Ao Sinus ED | 1.11 [1.09-1.13] | <0.001 | 1.13 [1.1-1.16] | <0.001 |
| Age |  |  | 1.07 [1.06-1.08] | <0.001 |
| Male sex |  |  | 0.72 [0.57-0.9] | 0.045 |
| BSA |  |  | 0.62 [0.36-1.06] | 0.653 |
| Hypertension |  |  | 0.89 [0.74-1.07] | 1.000 |
| Diabetes |  |  | 0.85 [0.62-1.14] | 1.000 |
| Ao STJ ED | 1.13 [1.1-1.16] | <0.001 | 1.14 [1.1-1.18] | <0.001 |
| Age |  |  | 1.07 [1.05-1.08] | <0.001 |
| Male sex |  |  | 0.85 [0.66-1.09] | 1.000 |
| BSA |  |  | 0.57 [0.31-1.03] | 0.572 |
| Hypertension |  |  | 0.9 [0.73-1.11] | 1.000 |
| Diabetes |  |  | 0.82 [0.58-1.15] | 1.000 |
| Ao Asc ED | 1.07 [1.04-1.1] | <0.001 | 1.07 [1.03-1.1] | 0.001 |
| Age |  |  | 1.07 [1.06-1.09] | <0.001 |
| Male sex |  |  | 0.93 [0.69-1.25] | 1.000 |
| BSA |  |  | 0.67 [0.32-1.41] | 1.000 |
| Hypertension |  |  | 0.73 [0.56-0.94] | 0.151 |
| Diabetes |  |  | 0.88 [0.57-1.31] | 1.000 |
| Mid-systolic aortic root measurements | | | | |
| Ao Ann MS | 1.14 [1.09-1.18] | <0.001 | 1.2 [1.13-1.28] | <0.001 |
| Age |  |  | 1.07 [1.06-1.08] | <0.001 |
| Male sex |  |  | 0.82 [0.65-1.03] | 0.855 |
| BSA |  |  | 0.69 [0.4-1.17] | 1.000 |
| Hypertension |  |  | 0.87 [0.72-1.05] | 1.000 |
| Diabetes |  |  | 0.87 [0.64-1.17] | 1.000 |
| Ao Sinus MS | 1.08 [1.05-1.1] | <0.001 | 1.07 [1.04-1.1] | <0.001 |
| Age |  |  | 1.08 [1.06-1.09] | <0.001 |
| Male sex |  |  | 0.91 [0.69-1.19] | 1.000 |
| BSA |  |  | 0.63 [0.34-1.19] | 1.000 |
| Hypertension |  |  | 0.82 [0.66-1.03] | 0.671 |
| Diabetes |  |  | 0.92 [0.64-1.3] | 1.000 |
| Ao STJ MS | 1.13 [1.09-1.16] | <0.001 | 1.14 [1.1-1.19] | <0.001 |
| Age |  |  | 1.07 [1.06-1.09] | <0.001 |
| Male sex |  |  | 0.86 [0.63-1.17] | 1.000 |
| BSA |  |  | 0.45 [0.21-0.95] | 0.380 |
| Hypertension |  |  | 0.86 [0.67-1.11] | 1.000 |
| Diabetes |  |  | 0.72 [0.44-1.12] | 1.000 |
| Ao Asc MS | 1.08 [1.05-1.12] | <0.001 | 1.07 [1.03-1.12] | 0.004 |
| Age |  |  | 1.07 [1.05-1.09] | <0.001 |
| Male sex |  |  | 0.91 [0.64-1.31] | 1.000 |
| BSA |  |  | 0.58 [0.23-1.41] | 1.000 |
| Hypertension |  |  | 0.91 [0.67-1.24] | 1.000 |
| Diabetes |  |  | 0,78 [0,45-1,3] | 1,000 |

P-Values were corrected for multiple testing using the post-hoc Holm method. *Ao = aortic, AR = aortic regurgitation, Asc = ascending, BSA = body surface area; CI = confidence interval, ED = end-diastolic, MS = mid-systolic, OR = odds ratio, STJ = sinotubular junction.*

Supplementary Table 3: Odds ratios and 95%-Cis for univariate and multivariate association of aortic root measurements with no vs. moderate/severe AR after exclusion of participants with mild AS

|  | Univariate | | Multivariate | |
| --- | --- | --- | --- | --- |
|  | OR [95%-CI] | p-value | OR [95%-CI] | p-value |
| End-diastolic aortic root measurements | | | | |
| Ao Ann ED | 1.12 [1.02-1.22] | 0.011 | 1.19 [1.06-1.34] | 0.01 |
| Age |  |  | 1.08 [1.06-1.11] | <0.001 |
| Male sex |  |  | 1.09 [0.67-1.76] | 1.0 |
| BSA |  |  | 0.22 [0.07-0.71] | 0.088 |
| Hypertension |  |  | 1.51 [0.99-2.39] | 0.359 |
| Diabetes |  |  | 0.92 [0.57-1.38] | 1.0 |
| Ao Sinus ED | 1.16 [1.12-1.2] | <0.001 | 1.2 [1.14-1.26] | <0.001 |
| Age |  |  | 1.06 [1.04-1.09] | <0.001 |
| Male sex |  |  | 0.76 [0.48-1.19] | 1.0 |
| BSA |  |  | 0.17 [0.06-0.47] | 0.008 |
| Hypertension |  |  | 1.81 [1.21-2.78] | 0.047 |
| Diabetes |  |  | 0.85 [0.54-1.26] | 1.0 |
| Ao STJ ED | 1.25 [1.18-1.31] | <0.001 | 1.27 [1.19-1.36] | <0.001 |
| Age |  |  | 1.07 [1.05-1.1] | <0.001 |
| Male sex |  |  | 0.85 [0.53-1.37] | 1.0 |
| BSA |  |  | 0.2 [0.06-0.64] | 0.059 |
| Hypertension |  |  | 1.53 [1-2.44] | 0.359 |
| Diabetes |  |  | 0.95 [0.59-1.44] | 1.0 |
| Ao Asc ED | 1.21 [1.15-1.26] | <0.001 | 1.21 [1.14-1.28] | <0.001 |
| Age |  |  | 1.07 [1.04-1.1] | <0.001 |
| Male sex |  |  | 0.84 [0.49-1.45] | 1.0 |
| BSA |  |  | 0.29 [0.08-1.07] | 0.259 |
| Hypertension |  |  | 1.66 [0.96-3.05] | 0.359 |
| Diabetes |  |  | 0.92 [0.53-1.48] | 1.0 |
| Mid-systolic aortic root measurements | | | | |
| Ao Ann MS | 1.16 [1.07-1.26] | 0.001 | 1.26 [1.12-1.41] | <0.001 |
| Age |  |  | 1.07 [1.05-1.1] | <0.001 |
| Male sex |  |  | 0.96 [0.62-1.5] | 1.0 |
| BSA |  |  | 0.26 [0.09-0.76] | 0.088 |
| Hypertension |  |  | 1.7 [1.14-2.62] | 0.11 |
| Diabetes |  |  | 0.84 [0.54-1.24] | 1.0 |
| Ao Sinus MS | 1.18 [1.13-1.22] | <0.001 | 1.22 [1.15-1.29] | <0.001 |
| Age |  |  | 1.07 [1.05-1.1] | <0.001 |
| Male sex |  |  | 0.68 [0.4-1.17] | 1.0 |
| BSA |  |  | 0.2 [0.06-0.69] | 0.088 |
| Hypertension |  |  | 1.71 [1.07-2.83] | 0.213 |
| Diabetes |  |  | 0.92 [0.54-1.44] | 1.0 |
| Ao STJ MS | 1.26 [1.19-1.33] | <0.001 | 1.34 [1.25-1.43] | <0.001 |
| Age |  |  | 1.07 [1.04-1.11] | <0.001 |
| Male sex |  |  | 0.55 [0.3-1] | 0.504 |
| BSA |  |  | 0.16 [0.04-0.65] | 0.088 |
| Hypertension |  |  | 2.01 [1.16-3.69] | 0.137 |
| Diabetes |  |  | 0.99 [0.54-1.64] | 1.0 |
| Ao Asc MS | 1.23 [1.16-1.3] | <0.001 | 1.26 [1.17-1.35] | <0.001 |
| Age |  |  | 1.08 [1.04-1.12] | <0.001 |
| Male sex |  |  | 0.7 [0.36-1.36] | 1.0 |
| BSA |  |  | 0.21 [0.04-1.08] | 0.259 |
| Hypertension |  |  | 1.4 [0.75-2.74] | 0.914 |
| Diabetes |  |  | 1.07 [0.53-1.92] | 1.0 |

P-Values were corrected for multiple testing using the post-hoc Holm method. *Ao = aortic, AR = aortic regurgitation, Asc = ascending, BSA = body surface area; CI = confidence interval, ED = end-diastolic, MS = mid-systolic, OR = odds ratio, STJ = sinotubular junction.*

Supplementary Table 4. P-Values for interaction of all aortic root components

|  | OR [95%-CI] | p-value |
| --- | --- | --- |
| Ao Ann ED | 1.07 [0.82-1.37] | 0.623 |
| Ao Sinus ED | 1.34 [1.05-1.72] | 0.019 |
| Ao STJ ED | 1.03 [0.81-1.32] | 0.826 |
| Ao Asc ED | 1.25 [1.05-1.48] | 0.010 |
| Ao Ann MS | 1.20 [0.91-1.59] | 0.212 |
| Ao Sinus MS | 0.75 [0.58-0.97] | 0.027 |
| Ao STJ MS | 1.11 [0.88-1.41] | 0.367 |
| Ao Asc MS | 0.95 [0.79-1.14] | 0.576 |

All aortic root measurements were included in the same regression analysis. *Ao = aortic, Asc = ascending, ED = end-diastolic, MS = mid-systolic, STJ = sinotubular junction.*

Supplementary Figure 1. Forest plots of multivariate logistic regression including end-diastolic aortic diameters

A. Aortic annulus, B. Aortic sinus, C. Aortic sintobuluar junction, D. Ascending aorta. P-Values were corrected for multiple testing using the post-hoc Holm method. *Ao = aortic, AR = aortic regurgitation, Asc = ascending, BSA = body surface area; CI = confidence interval, ED = end-diastolic, MS = mid-systolic, OR = odds ratio, STJ = sinotubular junction.*

Supplementary Figure 2. Forest plots of multivariate logistic regression including mid-systolic aortic diameters

A. Aortic annulus, B. Aortic sinus, C. Aortic sinotubular junction, D. Ascending aorta. P-Values were corrected for multiple testing using the post-hoc Holm method. *Ao = aortic, AR = aortic regurgitation, Asc = ascending, BSA = body surface area; CI = confidence interval, ED = end-diastolic, MS = mid-systolic, OR = odds ratio, STJ = sinotubular junction.*
